# Oral lipoma: Case report and review of literature

**DOI:** 10.1002/ccr3.2099

**Published:** 2019-03-14

**Authors:** Nima Dehghani, Farnoosh Razmara, Tahereh Padeganeh, Xaniar Mahmoudi

**Affiliations:** ^1^ Department of Oral and Maxillofacial Surgery Tehran University of Medical Sciences Tehran Iran; ^2^ School of Dentistry, International Campus Tehran University of Medical Sciences Tehran Iran

**Keywords:** benign bone tumor, intraoral lipoma, intraosseous lipoma, soft tissue tumor

## Abstract

Lipoma is a benign neoplasm that primarily affects the middle‐aged individuals and has a rare oral cavity occurrence. Given its noninvasive behavior and low recurrence rate, surgical conservative management should be regarded as the best therapeutic option. This paper highlights two patients along with their improved conditions following the treatment.

## 1 INTRODUCTION

### 1.1 General description

Oral lipoma, a benign tumor of mesenchymal origin, is composed of mature adipocytes and is usually separated by a thin fibrous connective tissue capsule.^1^, ^2^ Roux was the first to describe soft tissue lipoma in 1848 as a yellowish epulis,^3^ and Cornil and Ranvier presented the first case of intraosseous lipoma (IOL) in 1880.^4^ Most cases of oral lipoma are soft tissue lesions. About 15%‐20% of soft tissue lipomas occur in the head and neck area, of which only 1%‐4% are observed intraorally.^2^ The incidence of IOL is very low, about 0.1% and infrequently seen in the maxillofacial region.^5^ Oringer reported the first case of mandibular IOL in 1948.^2^

The incidence of this tumor appears to be related to the amount of adipose tissue due to the widespread area of the lesion. Lipoma is found more often in the buccal mucosa, which is full of adipose tissue owing to adjacency to the buccal fat pad.^6^, ^7^ Other sites where lipoma is common include the lips, tongue, floor of the mouth, palate, vestibule, mandible, and retromolar pad. On the other hand, the salivary glands, gingivobuccal fold, parotid, masseteric region and neck, and pharynx/larynx are less frequently involved.^8^, ^9^, ^10^ IOL often occurs in the metaphysis of long bones and the medullary bone of the calcaneus, the jaw being considered an uncommon location.^11^ Mandibular symphysis, body, and ramus are the most common locations for mandibular IOL. Maxillary involvement has also been reported.^4^, ^12^ Some scholars believe IOL may be associated with osteoporotic bone or ischemic trauma, but others see it as the beginning of a benign neoplasm.^5^

### 1.2 Etiology

The etiology of oral lipoma is unclear. Some studies have acknowledged that mechanical factors, endocrine system, inflammation, obesity, chromosomal abnormalities, radiation, trauma, mucosal infections, and chronic irritation can contribute to the development of oral lipoma.^13^

### 1.3 Histopathological features

The main findings of the histopathological view of both soft tissue and intraosseous lipomas are arrangements of mature adipocytes that are divided into lobules by the connective tissue septae. Usually, a thin fibrous capsule surrounds the tumor. Several types of soft tissue lipoma are described based on microscopic variations. The most common type is fibrolipoma, which is characterized by the presence of the fibrous components adjacent to the fat cells. Other types such as osteolipoma, chondrolipoma, intramuscular or infiltrating lipomas, salivary gland lipomas, pleomorphic lipomas, angiolipomas, myxoid lipomas, spindle cell lipomas, and atypical lipomas are scarce.^14^ Three stages of intraosseous lipoma are introduced based on the degree of involution: stage 1, lesions with no secondary necrosis; stage 2, lesions with partial necrosis; and stage 3, lesions with complete secondary necrosis.^15^

### 1.4 Clinical presentation

Most lipoma cases are adult patients aged 40‐60 years. These tumors are slow‐growing, painless, soft, circumscribed, and associated with submucosal nodules with either a sessile or a pedunculated base.^6^, ^16^ The color of oral lipomas varies from yellow to pink depending on the depth of the lesion,^9^ most of which are about 10 mm in diameter.^1^

Most cases of IOL are accidentally diagnosed during a radiographic examination. The symptoms of IOL are different depending on its size, position, evolution, and growth rate. This tumor may be associated with pain, swelling, and numbness.^17^, ^18^

Adequate surgical excision without a safe margin, which has a rare recurrence, is the treatment of choice for this tumor.^8^, ^19^

## 2 CASE PRESENTATION

### 2.1 Case 1

**Figure 1:**
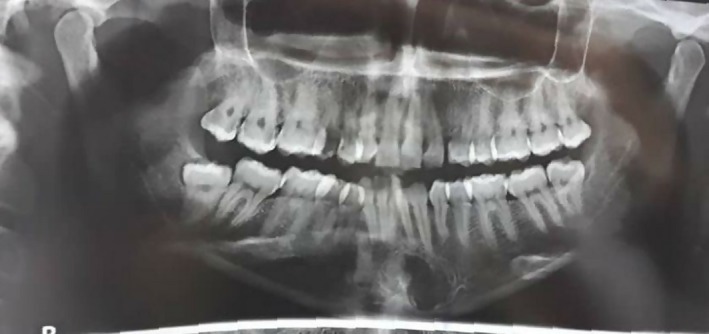
Radiograph of a patient with intraosseous lipoma. This is panoramic view which shows a unilocular lesion in the symphysis of the mandible

**Figure 2:**
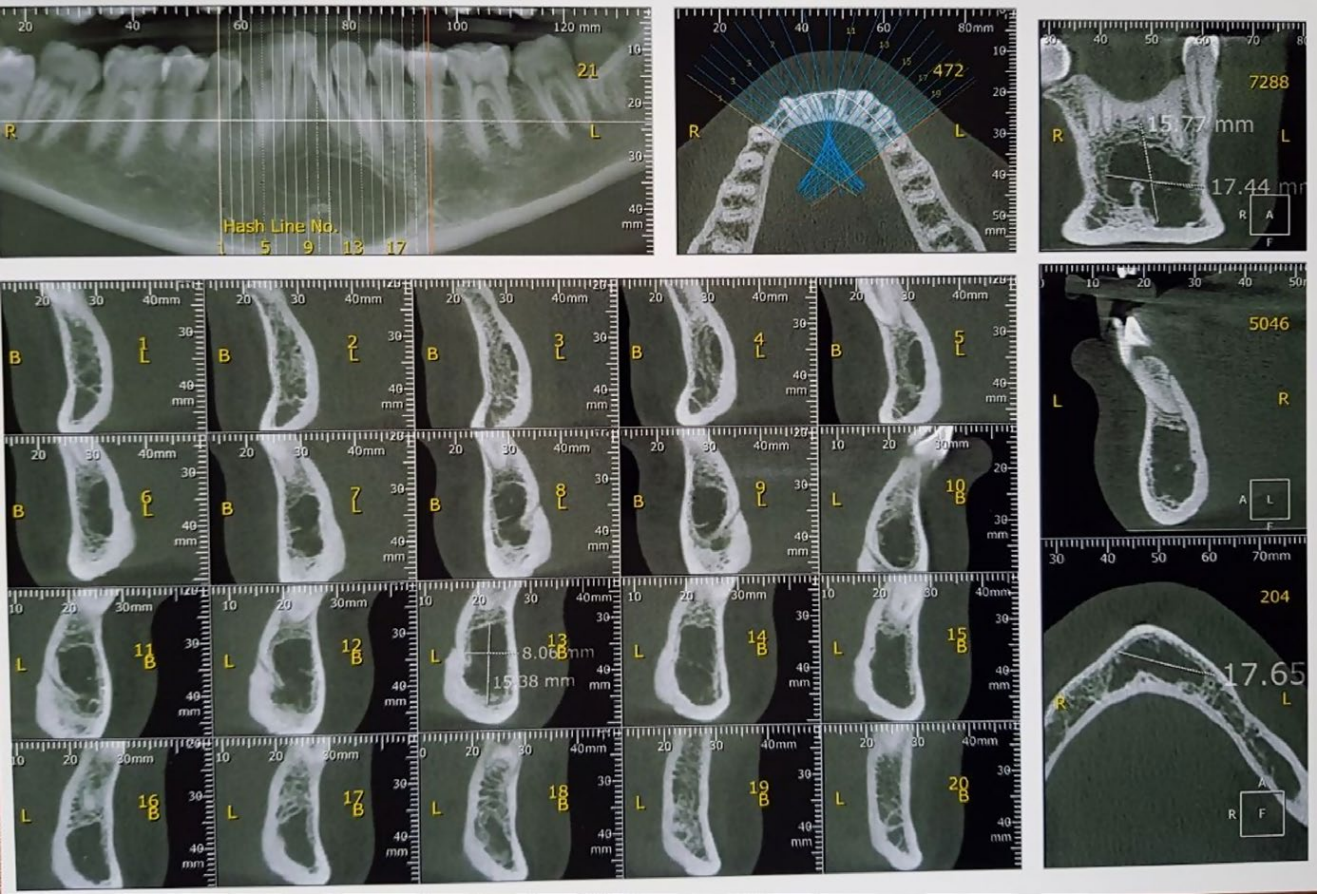
Radiograph of a patient with intraosseous lipoma. This is CBCT (cone beam computed tomography) view of the mandible, which shows a unilocular radiolucent lesion in the symphysis of the mandible

**Figure 3:**
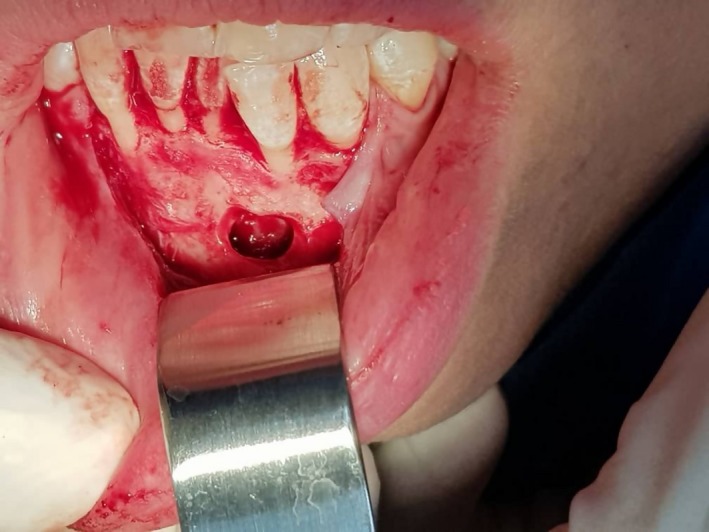
Intraoral photograph of a patient with intraosseous lipoma in the mandible during the excision of the lesion

**Figure 4:**
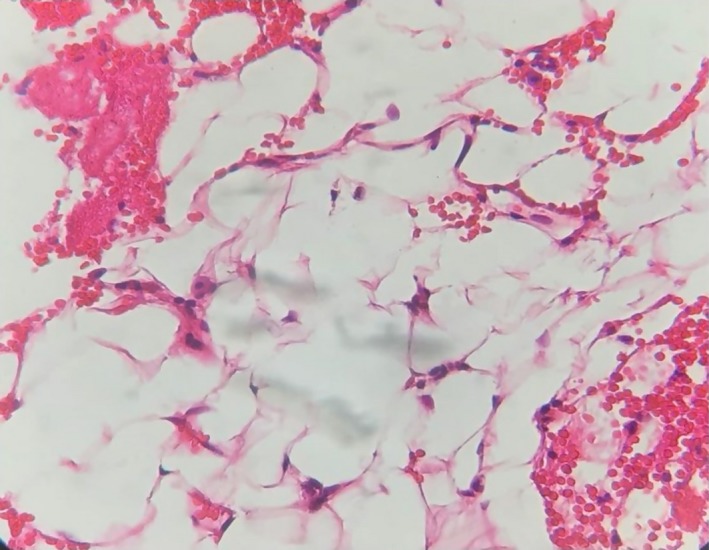
Histological view of intraosseous lipoma in the mandible

A 33‐year‐old woman referred to an orthodontist due to mandibular anterior crowding. While assessing her panoramic radiography (Figure 1), her dentist found a unilocular radiolucent lesion with a well‐defined sclerotic lesion that extended from the left mandibular canine to the right canine. She did not have a medical history of the disease. Intraoral and extraoral examinations were normal, and there were no expansion and pain in the palpation region. All mandibular anterior teeth were checked through electrical pulp testing, all of which were vital. The patient was referred to a maxillofacial surgeon for further evaluation. Cone beam computed tomography (CBCT) was requested for the patient. The radiographic examination showed a regular lesion border without any expansion in the buccal and lingual plates, root resorption, or root displacement (Figure 2). The lesion was biopsied. First, bilateral mental nerve block anesthesia was performed. Then, the envelope flap was raised between the first premolars and the bone was removed by a surgical bur. Next, the lesion was curetted and sent to a pathology center (Figure 3). The histopathological assessment showed a mature adipocyte with an area of hemorrhage but no atypical fat cell. Hence, it was found to be an IOL (Figure 4). In the follow‐up visits, there were no complications or recurrence, and the defect was healed properly.

### 2.2 Case 2

**Figure 5:**
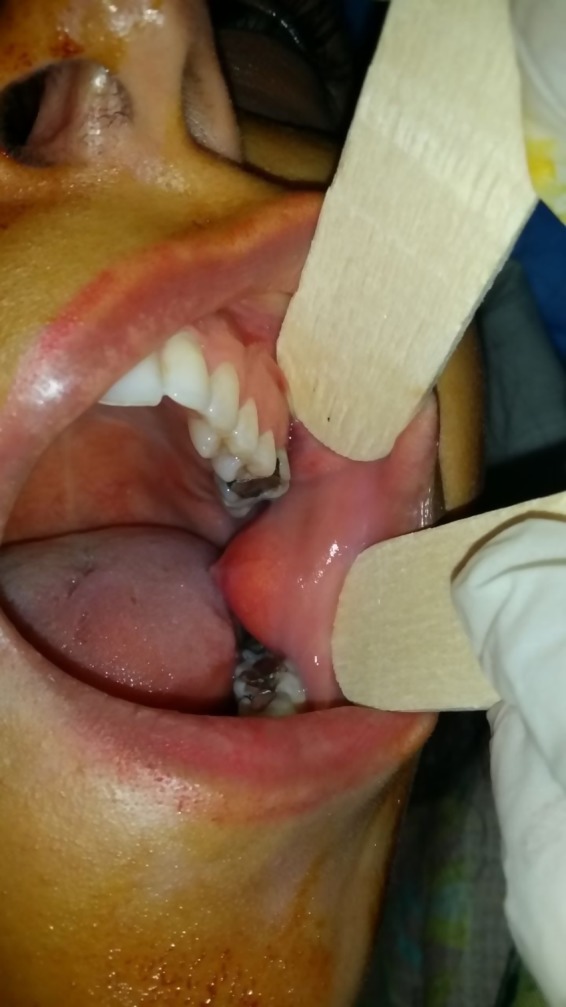
Intraoral view of soft tissue lipoma in buccal mucosa

**Figure 6:**
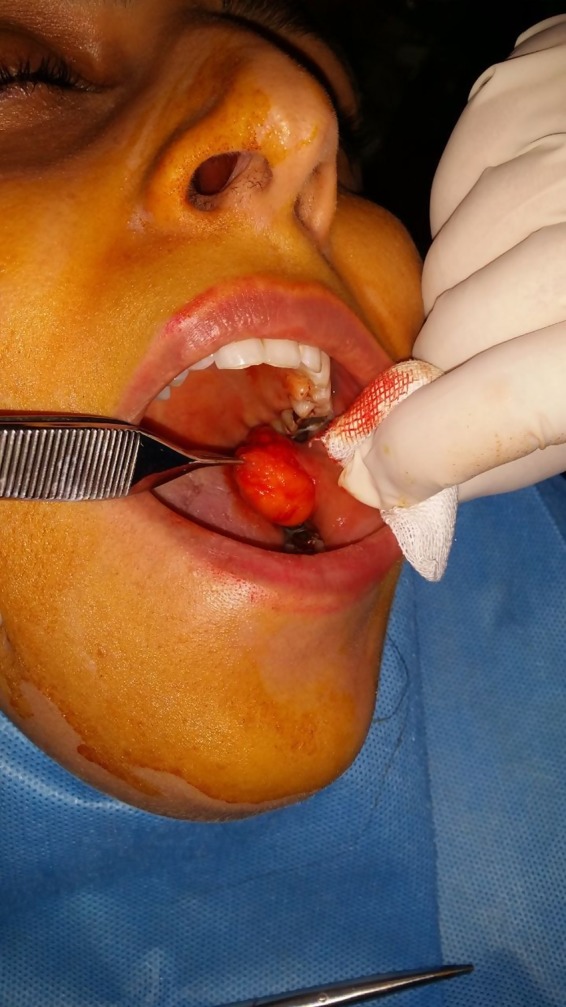
Clinical view of the oral lipoma in buccal mucosa during surgical excision of the lesion

**Figure 7:**
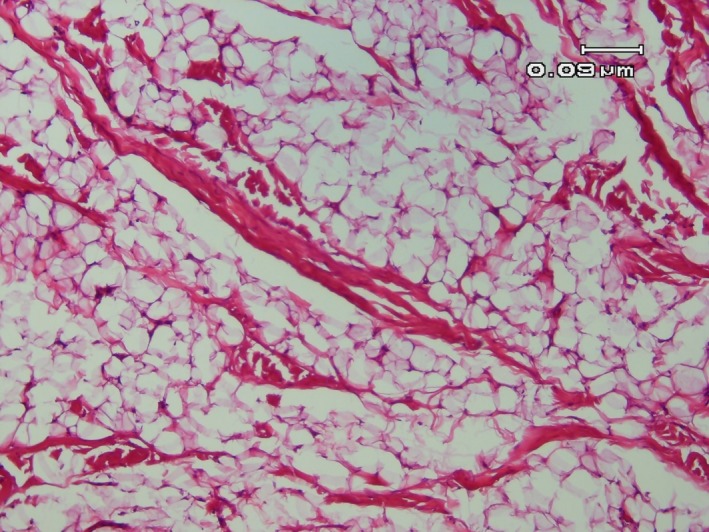
Histological view of the soft tissue lipoma, which shows an adipose tissue and a thin capsule surrounding the lesion with diagnosis of fibrolipoma

A 25‐year‐old woman referred to a maxillofacial department. Her chief complaint was painless swelling in the buccal mucosa for about 2 years, which interfered with her dental occlusion. The lesion was about 1.5 cm and mainly soft on palpation (Figure 5). Excisional biopsy was done under local anesthesia. The incision was about 2 cm and was inferior and parallel to the Stensen's duct (Figure 6). The lesion was capsulated and completely dissected. The laboratory examination revealed an adipose tissue and a thin capsule surrounding the lesion and pathologic diagnosis showed an intraoral fibrolipoma (Figure 7). There were no complications during and after the surgery and no sign of recurrence after 12 months.

## 3 MATERIALS AND METHODS

The present study reviewed the literature of the past 10 years on oral and intraosseous lipomas in the PubMed database (Table 1). The selection criteria included literature reviews, case series, and case reports in human in English language. The articles that did not contain useful information were eliminated. The papers contained information about sex, age, location, and histopathological pattern.

**Table 1:** Classification of intraoral lipoma based on the year of publication

Authors	Age	Sex	Site	Histopathological diagnosis	Treatment
Saghafi et al (2008)	68	M	Right mandibular alveolar mucosa	Osteolipoma	Surgical excision
Adoga AA et al (2008)	35	F	Left cheek	Lipoma	Surgical excision
Imai et al (2008)	72	M	Tongue	Spindle cell lipoma	Surgical excision
Altug HA et al (2009)	22	M	Cheek	Angiolipomas	Surgical excision
Kumaraswamy et al (2009)	30‐60 Avg: 45.8	3 M 1 F	Three in buccal mucosa, one in vestibule	Three lipomas, one fibrolipoma	Surgical excision
Cakarer S et al (2009)	45	F	Mandible	Intraosseous lipoma	Surgical excision
Jang YW et al (2009)	62	F	Submandibular gland	Sialolipoma	Surgical excision
Pusiol T et al (2009)	73	M	Submandibular gland	Sialolipoma	Surgical excision
Vecchio et al (2009)	52	M	Buccal mucosa	Spindle cell lipoma	Surgical excision
Nonaka et al (2009)	30	M	Tongue	Chondrolipoma	Surgical excision
De freitas et al (2009)	29‐91 Avg: 54.6	20 F 6 M	Nine in buccal mucosa, seven in tongue, four in the lower lip, three in the floor of the mouth, two in retromolar area, one in vestibule	Fifteen lipomas, seven fibrolipomas, two intramuscular lipomas, one spindle cell lipoma, one sialolipoma	Surgical excision
Okada H et al (2009)	66	F	Hard palate	Sialolipoma	Surgical excision
De Moraes M et al (2010)	72	F	Hard palate	Sialolipoma	Surgical excision
Brkic et al (2010)	59	F	Buccal mucosa	Angiofibrolipoma	Surgical excision
De castro et al (2010)	47	F	Left cheek	Osteolipoma	Surgical excision
SY et al (2010)	47	F	Soft palate	Lipoma	Surgical excision
Manjunatha et al (2010)	55‐75 Avg: 66.6	3 M	Buccal mucosa	Fibrolipoma	Surgical excision
Diom es et al (2010)	21	F	Parotid region	Osteolipoma	Surgical excision
Gonzalez‐Perez et al (2010)	61	F	Left mandibular ramus	Intraosseous lipoma	Surgical excision
Studart‐Soares et al (2010)	21‐73 Avg: 53.4	6 F 4 M	Five in buccal mucosa, three in vestibule one in gingiva, one in retromolar area	Four lipomas, four fibrolipoma, one angiolipoma, one myxolipoma	Surgical excision
Hoseini et al (2010)	50‐63	2 M	One in tongue, one in palate	Lipoma	Surgical excision
Venkateswarlu et al (2011)	6	M	Retromolar region	Lipoma	Surgical excision
Morais (2011)	45	F	Mandible	Intraosseous lipoma	Resection
Brucoli et al (2011)	43	M	Right cheek	Lipoma	Surgical excision
Ono S et al (2011)	52	M	Tongue	Myxolipoma	Surgical excision
Martinez‐Mata G et al (2011)	12	F	Right cheek	Angiomyxolipoma	Surgical excision
Taira et al (2011)	65	F	Right mandibular gingiva	Lipoma	Surgical excision
Akrish S et al (2011)	52	M	Submandibular gland	Sialolipoma	Surgical excision
Akrish S et al (2011)	67	F	Palate	Sialolipoma	Surgical excision
Nonaka et al (2011)	27‐73 Avg: 58.3	3 F 1 M	One in tongue, one in buccal mucosa, one in the floor of the mouth, one in retromolar area	Sialolipoma	Surgical excision
Santos et al (2011)	58	M	Buccal mucosa	Lipoma	Surgical excision
Adebiyi KE et al (2011)	37	F	Palate	Osteolipoma	Surgical excision
Motagi et al (2012)	36	M	Right buccal mucosa	Lipoma	Surgical excision
Binmadi et al (2012)	54	F	Lower lip	Sialolipoma	Surgical excision
Lee et al (2012)	71	M	Tongue	Lipoma	Surgical excision
Khubchandani et al (2012)	10	F	Buccal mucosa	Fibrolipoma	Surgical excision
Qayyum S et al (2013)	69	M	Parotid	Sialolipoma	Surgical excision
D'Antonio A et al (2013)	44	M	Parotid gland	Spindle cell lipoma	Surgical excision
Sun Z et al (2013)	48	M	Chin	Ossifying parosteal lipoma	Surgical excision
Basher (2013)	15	M	Anterior area	Intraosseous lipoma	Resection
Kiran A et al (2013)	53	F	Right cheek	Lipoma	Surgical excision
Pattipati et al (2013)	37	M	Palate	Lipoma	Surgical excision
Junior et al (2013)	64	F	Tongue	Spindle cell lipoma	Surgical excision
Chandak et al (2013)	75	M	Tongue	Lipoma	Surgical excision
Kumar et al (2014)	77	M	Lower left mental region	Lipoma	Surgical Excision
Tsumuraya et.al (2014)	58	M	Cheek	Intramuscular lipoma	Surgical excision
Raj AA et al (2014)	72	M	Floor of the mouth	Lipoma	Surgical excision
Raj V et al (2014)	35	M	Tongue	Chondrolipoma	Surgical excision
Fomete et al (2014)	50	F	Tongue	Neurofibrolipoma	Surgical excision
Kamakshi et al (2014)	6	F	Lower lip	Chondrolipoma	Surgical excision
Amaral et al (2015)	51	M	Left cheek	Osteolipoma	Surgical excision
Raghunath et al (2015)	20	F	Floor of the mouth	Osteolipoma	Surgical excision
Castellani et al (2015)	25	F	Right mandibular ramus	Fibrolipomas	Surgical excision
Baonerkar et al (2015)	63	M	Tongue	Lipoma	Surgical excision
Stoopler (2015)	53	F	Buccal mucosa	Fibrolipomas	Surgical excision
Jaeger et al (2015)	56	M	Hard palate	Spindle cell lipoma	Surgical excision
Hancer et al (2015)	31	F	Hard palate	Spindle cell lipoma	Surgical excision
Jun choi et al (2016)	68	M	Nearby mental foramen	Lipoma	Surgical excision
Tom (2016)	15	F	Cheek	Lipoma	Surgical excision
Baykul T et al (2016)	44	M	Parotid gland	Lipoma	Parotidectomy
Shin et al (2016)	39	F	Coronoid process	Intraosseous lipoma	Osteotomy
Lu SL et al (2016)	78	M	Tongue	Lipoma	Surgical excision
Lwase et al (2016)	71	M	Left buccal mucosa	Fibrolipomas	Surgical excision
Raviraj et al (2016)	38	F	Left cheek	Osteolipoma	Surgical excision
Sharma et al (2016)	32	M	Left mandibular posterior region	Lipoma	Surgical excision
Mehendirratta et al (2016)	60	M	Mandibular buccal vestibule	Lipoma	Surgical excision
Seelam et al (2016)	55	F	Right retromolar region	Osteolipoma	Surgical excision
Waskowska et al (2017)	32	M	Body of the mandible	Intraosseous lipoma	Surgical excision
Cooper et al (2017)	53	M	Right mandibular body	Intraosseous spindle cell	Surgical excision
Coelho et al (2017)	78	M	Left buccomasseteric region	Lipoma	Surgical excision
Ohyama et al (2017)	4 mo	M	Hard palate	Lipoma	Surgical excision
Sanjuan et al (2017)	50	F	Left mandibular ramus and condyle	Intraosseous lipoma	Surgical excision and curettage
Tabakovic et al (2017)	43	F	Maxillary tuberosity	Intraosseous lipoma	Surgical excision
Bajpai M et al (2017)	51	M	Tongue	Angiomyxolipoma	Surgical excision
Arakeri et al (2018)	1	M	Parotid gland	Sialolipoma	Parotidectomy
Phulari et al (2018)	16	F	Left retromolar region	Fibrolipomas	Surgical excision
Phulari et al (2018)	60	M	Right first molar	Fibrolipomas	Surgical excision

In the present review, the authors presented two cases of oral lipoma.

## 4 RESULTS

A large number of articles were found, from which 77 articles were selected after applying the selection criteria (Table 1). Among the 120 cases, 58 cases were found in men (48.33%) and 62 cases (51.66%) in women. The average age was found to be 47.69 years, indicating that most lesions occurred in the 4th and 5th decades of life.

Buccal mucosa was the most common region for the occurrence of oral soft tissue lipoma, and mandibular body was the most common site for oral intraosseous lipoma. Simple lipoma was the most common histopathological pattern in oral soft tissue lipoma. Most authors considered surgical technique as a definitive treatment.

## 5 DISCUSSION

Lipoma is a benign tumor that can occur in any part of the body. Lipoma can be found in both soft and bony tissues.

The clinical features of intraoral lipoma can be related to the location of the lesion. They often refer to slow‐growing tumors associated with fatty tissue and vary in diameter, which contributes to the possibility of misdiagnosis.^20^

There are different reports about the relationship between lipoma and sex. The incidence of oral lipoma has been reported to be identical in the males and females, or male prevalence has been emphasized or vice versa.^21^

Bone lesions are often discovered by accident. Radiographic images show unilocular or multilocular radiolucent lesions with a honeycomb or soap bubble appearance and often an osteosclerotic border.^22^, ^23^ A definitive IOL cannot be diagnosed by a radiographic image. Computed tomography (CT) and magnetic resonance imaging (MRI) can detect these tumors easily. Despite the availability of all these techniques, histopathology remains the gold standard for diagnosis of lipomas.^24^

The differential diagnosis of IOL includes simple cyst, post‐traumatic cyst, aneurysmal bone cyst, giant cell granuloma, ameloblastoma, osteoblastoma, arteriovenous malformations, hemangiomas, infarcted bone, chondrosarcoma, and liposarcoma.^25^ The differential diagnosis of intraoral lipoma consists of oral dermoid and epidermoid cysts, oral lymphoepithelial cyst, benign salivary gland tumor, mucocele, benign mesenchymal neoplasm, ranula, ectopic thyroid tissue, and lymphoma. Lesions appearing as swelling on the dorsum of the tongue usually mimic hemangioma, lymphangioma, rhabdomyoma, neuroma, and neurofibroma.^26^

Complete surgical excision is the main treatment of lipoma. There is no recurrence after adequate excision. Injectable steroids are used to manage soft tissue lipoma, which can cause the atrophy of the adipose tissue and reduce the size of the tumor. Monthly injection of 1:1 mixture of lidocaine and triamcinolone acetonide is recommended to be administered to the center of the lesion.^26^

## CONFLICT OF INTEREST

None declared.

## AUTHOR CONTRIBUTION

ND, FR, TP and XM: involved in the conception and design of the work, data collection, drafting of the manuscript, critical revision of the manuscript, and final approval of the version to be published.

## References

[1] Egido‐Moreno S , Lozano‐Porras AB , Mishra S , Allegue‐ Allegue M , Marí‐Roig A , López‐López J . Intraoral lipomas: Review of literature and report of two clinical cases. J Clin Exp Dent. 2016;8:597‐603. 10.4317/jced.52926 PMC5149098 27957277

[2] Mehendirratta M , Jain K , Kumra M , Manjunatha BS . Lipoma of mandibular buccal vestibule: a case with histopathological literature review. BMJ Case Rep. 2016;2016:bcr2016215586. 10.1136/bcr-2016-215586 PMC4986158 27489068

[3] Roux M . On exostoses: there character. Am J Dent Sci. 1848;9:133‐134. PMC6059642 30749544

[4] Barker GR , Sloan P . Intraosseous lipomas: clinical features of a mandibular case with possible aetiology. Br J Oral Maxillofac Surg. 1986;24:459‐463. 2947620 10.1016/0266-4356(86)90063-x

[5] Basheer S , Abraham J , Shameena PM , Balan A . Intraosseous lipoma of mandible presenting as a swelling. J Oral Maxillofac Pathol. 2013;17:126‐128. 23798846 10.4103/0973-029X.110705 PMC3687168

[6] Manor E , Sion‐Vardy N , Joshua BZ , Bodner L . Oral lipoma: analysis of 58 new cases and review of the literature. Ann Diagn Pathol. 2011;15:257‐261. 21447447 10.1016/j.anndiagpath.2011.01.003

[7] Furlong MA , Fanburg‐Smith JC , Childers EL . Lipoma of the oral and maxillofacial region: Site and subclassification of 125 cases. Oral Surg Oral Med Oral Pathol Oral Radiol Endod. 2004;98:441‐450. 15472660 10.1016/j.tripleo.2004.02.071

[8] Fregnani ER , Pires FR , Falzoni R , Lopes MA , Vargas PA . Lipomas of the oral cavity: clinical findings, histological classification and proliferative activity of 46 cases. Int J Oral Maxillofac Surg. 2003;32:49‐53. 12653233 10.1054/ijom.2002.0317

[9] Agha‐Hosseini F , Moslemi E . Angiofibrolipoma of the retromolar pad region: case report. N Y State Dent J. 2014;80:33‐37. 25219062

[10] Taira Y , Yasukawa K , Yamamori I , Iino M . Oral lipoma extending superiorly from mandibular gingivobuccal fold to gingiva: a case report and analysis of 207 patients with oral lipoma in Japan. Odontology. 2012;100:104‐108. 21607594 10.1007/s10266-011-0027-0

[11] Kapakuya A , Subasi M , Dabak N , Ozkul E . Osseous lipoma: eleven new cases and review of the literature. Acta Orthop Belg. 2006;72:603‐614. 17152426

[12] Keogh PV , McDonnell D , Toner M . Intraosseous mandibular lipoma: a case report and review of the literature. J Ir Dent Assoc. 2004;50:132‐134. 15481526

[13] Cocca S , Viviano M , Parrini S . Unusual complications caused by lipoma of the tongue. J Kor Assoc Oral Maxillofac Surg. 2017;43:S6‐S8. 10.5125/jkaoms.2017.43.S1.S6 PMC5770474 29354591

[14] Fregnani ER , Pires FR , Falzoni R , Lopes MA , Vargans PA . Lipomas of the oral cavity: clinical findings, histological classification and proliferative activity of 46 cases. Int J Oral Maxillofac Surg. 2003;32:49‐53. 12653233 10.1054/ijom.2002.0317

[15] Milgram JW . Intraosseous lipomas: radiologic and pathologic manifestations. Radiology. 1988;167:155‐160. 3347718 10.1148/radiology.167.1.3347718

[16] Ono S , Rana M , Takechi M , et al. Myxolipoma in the tongue – a clinical case report and review of the literature. Head Neck Oncol. 2011;3:50. 22185472 10.1186/1758-3284-3-50 PMC3259069

[17] Gonzalez‐Perez LM , Pérez‐Ceballos JL , Carranza‐Carranza A . Mandibular intraosseous lipoma: clinical features of a condylar location. Int J Oral Maxillofac Surg. 2010;39:617‐620. 20188513 10.1016/j.ijom.2010.01.011

[18] Darling MR , Daley TD . Radiolucent lesion of the anterior mandible. Oral Surg Oral Med Oral Pathol Oral Radiol Endod. 2005;99:529‐531. 15829871 10.1016/j.tripleo.2004.12.022

[19] Trandafir D , Gogalniceanu D , Trandafir V , Caruntu ID . Lipomas of the oral cavity: a retrospective study. Rev Med Chir Soc Med Nat Iasi. 2007;111:754‐758. 18293712

[20] Debnath SC , Saikia A . Lipoma of the parotid gland extending from the superficial to the deep lobe: a rarity. Br J Oral Maxillofac Surg. 2010;48:203‐204. 19910088 10.1016/j.bjoms.2009.07.028

[21] Karakosta P , Matiakis A , Anagnostou E , Kololotronis A . Oral lipoma located at the left lower vestibule‐ report of a case and a brief review of the literature. Balkan J of Dent Med. 2018;22:49‐52.

[22] Waskowska J , Wojcik S , Koszowski R , Drozdzoska B . Intraosseous lipoma of the mandibula: a case report and review of the literature. Open Med Wars. 2017;12:45‐49. 28401200 10.1515/med-2017-0008 PMC5385973

[23] González‐Pérez LM , Pérez‐Ceballos JL , Carranza‐Carranza A . Mandibular intraosseous lipoma: clinical features of a condylar location. Int J Oral Maxillofac Surg. 2010;39:617‐620. 20188513 10.1016/j.ijom.2010.01.011

[24] Epivatianos A , Markopoulos AK , Papanayotou P . Benign tumors of adipose tissue of the oral cavity: a clinicopathologic study of 13 cases. J Oral Maxillofac Surg. 2000;58:1113‐1117. 11021705 10.1053/joms.2000.9568

[25] Sanjuan A , Dean A , Garcia B , Alamillos F , Roldan E , Blanco A . Condylar intramedullary intraosseous lipoma: Contribution of a new case and review of the literature. J Clin Exp Dent. 2017;9:e498‐e502. 28298998 10.4317/jced.53421 PMC5347305

[26] Surej Kumar LK , Kurien NM , Raghavan VB , Varun Menon P , Khalam SA . Lipoma: a case report. Case Rep Med. 2014;2014;480130. 24592278 10.1155/2014/480130 PMC3926394

